# Addressing patients’ unmet needs related to multidrug-resistant tuberculosis (MDR-TB) care: A qualitative research study from Pune city, India

**DOI:** 10.1371/journal.pone.0295508

**Published:** 2023-12-28

**Authors:** Sachin Atre, Jayshri Jagtap, Mujtaba Faqih, Yogita Dumbare, Trupti Sawant, Sunil Ambike, Maha Farhat

**Affiliations:** 1 Dr. D.Y. Patil Medical College, Hospital and Research Centre Dr. D.Y. Patil Vidyapeeth, Pune, India; 2 HaystackAnalytics Pvt Ltd, Mumbai, India; 3 Department of Biomedical Informatics, Harvard Medical School, Boston, Massachusetts, United States of America; 4 Division of Pulmonary and Critical Care, Massachusetts General Hospital, Boston, Massachusetts, United States of America; Stellenbosch University, SOUTH AFRICA

## Abstract

**Aim:**

We aimed to identify and describe the unmet needs of patients with multidrug-resistant tuberculosis (MDR-TB).

**Methods:**

As a part of larger cross-sectional mixed-methods (qualitative and quantitative data) study on pathways to MDR-TB care, here we present the qualitative component. We interviewed 128 (56 men and 72 women) individuals who had MDR-TB, aged > = 15 years, registered and treated under the National TB Elimination Program (NTEP) in Pune city of India. We carried out thematic analysis of participants’ narratives.

**Results:**

We found that delays in diagnosis, lack of counseling, late referral to the NTEP and unwarranted expenditure were the main barriers to care that study participants experienced in the private sector. Provider dismissal of symptoms, non-courteous behavior, lack of hygiene in the referral centers, forced stay with other patients and lack of support for psychological/psychiatric problems were identified as a few additional challenges that participants faced at the NTEP care centers.

**Conclusion:**

Using qualitative data from experiences of participants with MDR-TB, we identify patients’ several unmet needs, attention to which can improve MDR-TB care. Educating private providers about MDR-TB risk and available rapid molecular assays can help the timely diagnosis of MDR-TB and reduce patients’ out of pocket costs. At the RNTCP/NTEP, measures such as training health workers to build rapport with patients, maintaining hygienic environments in the health centers with adequate isolation of participants with MDR from other serious cases, referral of patients with psychiatric symptoms to mental health specialists and monitoring drug shortages can help in improving care delivery.

## Introduction

Ram narrated his pathway to TB care which spanned over two years.

“I have been sick for the last two years. Initially, I had fever, weakness, and loss of appetite. I approached our family doctor several times over a 2-month period, but he could not diagnose the problem. He gave me medicines for a week each time I visited him. Then I went to a private hospital where the doctor advised me to get an X-ray (chest radiograph). I spent 300 rupees (~$4) and after the report was out, I was told that I had TB. I was told that I need to get ‘admitted to the hospital for saline’ but the bed charges would be 2000 rupees (US $24) per day in addition to the cost of the medicines. Since this expense was unaffordable to me, I returned to my family doctor. Later I was referred to another private hospital where my 6-month treatment for TB started. After having two rounds of treatment (6 months each), I did not feel better. Then I saw a chest specialist. I spent nearly 1.5 lakh rupees (US $1807) on treatment at these private hospitals. The chest specialist told me to get different tests done. But my uncle who had TB before, advised me that I should go for treatment from the government centre. So, I got admitted here at the health centre and I was told I have MDR-TB”.

Ram’s case is a typical presentation of an individual with TB in an urban Indian setting. It depicts a cascade of care that begins with the first help at a private provider. His narrative provides an example of the circumstances that force patients to change providers before finally converging on diagnosis and treatment in the public sector. This narrative also indicates the possible factors that are responsible for delays in the diagnosis of drug resistant forms of TB. It raises questions about TB care in the private sector and associated financial hardship.

Multidrug-resistant tuberculosis (MDR-TB) results from infection with TB bacilli resistant to the two most potent first-line anti-TB drugs- rifampicin and isoniazid. There are annually half a million incident cases of rifampicin resistant (RR) TB and 83% of those are MDR [1]. India is estimated to carry a fourth of the global burden of TB and MDR-TB [2]. Since 2014, the World Health Organization (WHO) recommends the use of rapid Nucleic Acid Amplification tests (NAATs) for detecting TB and rifampicin resistance [3]. In 2021, only three fourth of TB suspects from India received a NAAT detecting only 40% of the estimated patients with MDR/RR [3]. Of those, 90% were started on MDR-TB treatment, however the treatment success rate remains suboptimal at 57%. These data warrant a qualitative inquiry into patient perspectives and experiences to elucidate factors underlying challenges facing MDR-TB diagnosis and treatment.

Previous qualitative studies among participants with MDR-TB reported loss of financial resources before beginning of the treatment, long waiting hours in clinics, lack of access to health care facility, social stigma, misconceptions, high pill burden and severe side-effects of treatment as major barriers to care [4–7]. While these studies provide insights into some barriers that patients with MDR/DR-TB faced, they were limited by small samples and the study of specific settings that may not allow generalization. Since NAATs were more recently (since 2018), rolled out into care in the NTEP in Maharashtra, we aimed to study patient experiences and unmet needs as they navigate care for MDR TB. A qualitative analysis can identify barriers to access to NAAT based diagnosis and inform the delivery of timely and patient-centred care.

As a part of a larger mixed-methods study on pathways to TB care in Pune city of Maharashtra State in India, here we present the qualitative component with a patients’ perspective to complement the previously published quantitative component [8].

## Methods

### Setting

**Fig 1:**
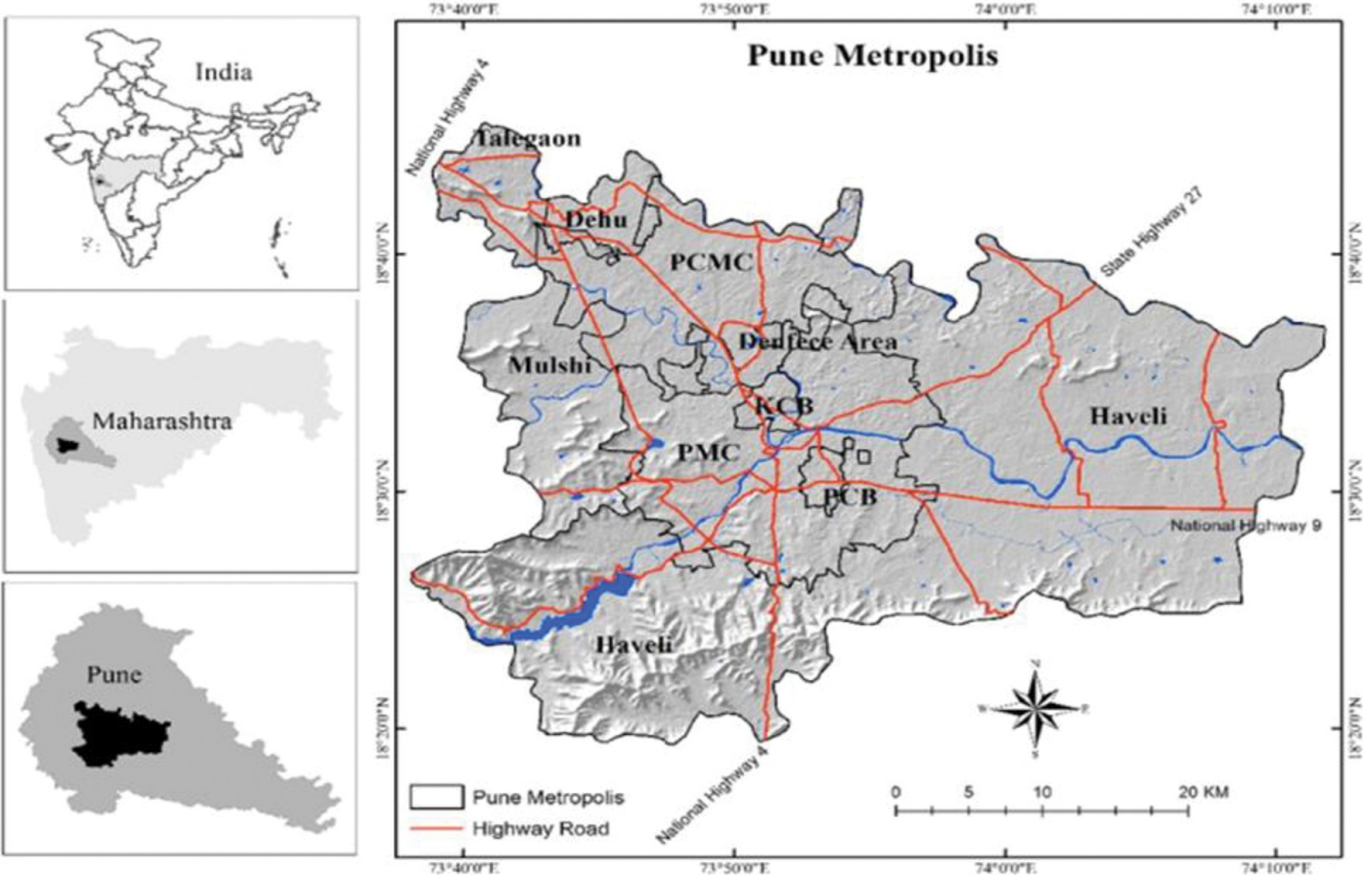
Pune metropolis.

The study was conducted between January 2018 and September 2019 in Pune Municipal Corporation (PMC) and Pimpri-Chinchwad Municipal Corporation (PCMC) of Maharashtra- the second most populous state in India. PMC and PCMC together cover a population of approximately 7 million (Population 5,057,709 as per the Census 2011) [9]. The city proper has a population density of 5,600 people per square kilometer (15,000/square mile) (Fig 1) [10]. The literacy rate in Pune is 89% and 62% of the population is under 30 years with a larger-than-average share of people in the 25–34 age group.

### Participants

As a part of a larger cross-sectional mixed-methods study on assessing pathways to TB care, we interviewed patients registered and treated under the RNTCP in PMC and PCMC area. We attempted to reach all patients registered for MDR-TB treatment in 15 RNTCP TB units (1 TB Unit covers 0.3–0.5 million population) (TUs) during the study period. We included individuals who were 15 years or older and had completed ≥ 3 months of second line anti-TB treatment to explore the issues that participants experienced during their course of treatment and to minimize the interviewers’ risk of exposure to infection. We interviewed participants aged 15–18 years along with one of their parents or guardians after seeking their consent. During the study period, 393 patients with MDR-TB were registered with the RNTCP in the study areas. As per the inclusion criteria, the study team approached 238 patients who were available at that time and enrolled 128 and excluded 110. Major reasons for exclusion were no phone contact/no response (46%); not interested in participation (28%); death before approach (15%); moved out of Pune (9%) and 2% had psychiatric symptoms. We could enrol 128 (56 men and 72 women) participants who consented for an in-depth interview (~33% of the total sample). Of 128 participants, 72% were young adults aged 15–35 years and remaining were aged 36–56 years. Fifty six percent were women, 62% were married. About 60% participants had school education up to secondary school or below. Seventy percent participants did not have own source of income as they were either unemployed or housewives/students. Nearly three-fourths of the participants were residents in crowded localities such as slums or informal settlements (Table 1).

**Table 1:** Socio-demographic characteristics of study participants.

Characteristics	MDR (n = 128)	
** **	**n**	**%**
**Age group (yrs.)**		
15–35	92	71.9
36–56	35	27.3
56+	1	0.8
**Gender**		
Male	56	43.8
Female	72	56.2
**Marital status**		
Unmarried	41	32.0
Married	80	62.5
Other (Widowed/ Separated)	7	5.4
**Family type**		
Nuclear	69	53.9
Joint and extended	55	42.9
Single	2	1.6
Stay with friends	2	1.6
**Education**		
Illiterate	5	3.9
Primary (1st -4th)	13	10.2
Secondary (5th-10th)	58	45.3
High secondary (11th/12th)	25	19.5
Graduation/Post graduation	27	21.1
**Occupation**		
Unemployed	35	27.3
Housewife	32	25.0
Student	23	18.0
Skilled laborer	15	11.7
Other	23	17.9
**Type of House/Locality**		
Crowded	97	75.7
Non-crowded	31	24.3

### Procedures

The study was approved by Harvard Medical School’s Institutional Review Board (IRB), the Institutional Ethics Committee of Dr. D.Y. Patil Vidyapeeth, the Indian Ministry of Health and Family Welfare, Central TB Division, and Maharashtra State TB office. The study team contacted participants registered in the TUs by phone if they met the above-described inclusion criteria. After obtaining the written consent, participants were interviewed using a structured set of questions that covered socio-demographic profile, time to TB diagnosis for the current episode and other details. Following the structured interview, participants were asked to return for an in-depth interview when the patient’s pathway to TB care were discussed in detail which included the reasons for help seeking, expenditure incurred, side effects of treatment, experience with the public (RNTCP) or private health system, problems faced because of TB etc. The study team conducted in-depth interviews in Marathi or Hindi as per the preference of the participants and e-recorded them. They transcribed those interviews in the local language and later translated into English.

### Analysis

The quantitative data from the survey were entered in Qualtrics and processed using MS-Excel. The main findings from the survey data are reported elsewhere [8], however some are described here again to contextualise and complement the qualitative data. The transcripts from in-depth interviews were analyzed using the MAXQDA software (VERBI, Version 18.0). Our analysis is based on the theoretical concept of ‘Structural Violence’-the term originally coined by John Galtung during the 1960s [11]. It relates to social structures—economic, political, legal, religious, and cultural—that stop individuals, groups, and societies from reaching their full potential. The concept was further adapted by Farmer et al. who showed through their work that mere structural changes to prevent epidemics would not suffice, but an important part is also to make the health care available for those who are already ill [12]. Our analysis is geared toward addressing the structural violence that patients with MDR-TB experienced while accessing the health services as evident from their own narratives and what possible solutions could be offered to improve the health care delivery. For the qualitative data analysis, we adapted Braun and Clarke’s thematic analysis method [13]. It is an iterative process consisting of following six steps: (1) Data familiarization: At the first step, we imported all the transcripts into MAXQDA. Each transcript was read by two investigators. The textual data (participants’ responses) were arranged as per the interview guide to maintain a logical flow. (2) Generating codes: After discussing with the study team, we generated a primary coding list in English, based on the questions in the interview guide. Further, based on the data, we generated some secondary codes and additional codes for the responses beyond questions in the interview guide. Two study team members coded narratives through an interrater coding. (3) Generating themes: The team identified most reported issues (unmet needs) by activating relevant codes and text records. (4) Reviewing themes: After reviewing retrieved narrative segments, common themes were identified. (5) Defining and naming themes: We named most observed themes. (6) Locating exemplars: Finally, we selected respondents’ narratives that reflected a particular theme as an example.

Since Indian health system consists of a large weakly regulated private sector and the public sector/NTEP and patients are known to visit both, we adapted a framework to analyse our data with reference to these two sectors. We identified following major themes: In the private sector: Low suspicion of MDR-TB and resulting diagnostic delays, lack of counselling, unwarranted expenditure, and private practitioners’ reluctance for referral to the RNTCP. In the RNTCP/public sector, we identified non-courteous behaviour, unclean environment in the referral centres, unwilling contact with other drug resistant patients, problems faced due to injectable, barriers in accessing medications and lack of support for mental health problems such as depression and suicidal tendency. We first provided the quantitative data to present the magnitude/the extent of that unmet need and supplemented it with the narratives to explain its nature.

## Results

### Experiences within the private sector

#### Low suspicion of MDR-TB and diagnostic delays

Findings from the published survey data [8] indicate that 68% participants sought first help from the private sector. However, only 7% of participants received their diagnosis as MDR-TB in the private sector; 49% (63 of 128) of participants reported no history of TB, suggesting that they had an infection with a primary MDR-TB strain, but were not even suspected. The median duration between the symptom onset to first help seeking was 15 days (IQR 9–30 days), whereas the median duration between the first encounter and diagnosis of MDR-TB was higher [80 days (IQR 44–161)]. This indicates that the major diagnostic delays happened after the patient visited the first provider, which was the private provider for a large proportion of participants. The median total number of providers visited before diagnosis was 4 (IQR, 3–5). Narratives from the participants elaborated their experiences in the private sector.

“In the month of May, I used to have fever and loss of appetite. I visited a couple of doctors whom I generally visit for any health problem. The first one gave me medicines for a week. Later he put me on saline. My blood was also tested, but he could not diagnose the illness. Then I went to another doctor, who again advised blood tests and X-ray. I was given medicines for cough. The fever used to go and come again. Then, in the month of June, I went to my village. I was unwell so I was admitted there and given saline. Then I was admitted in ‘X’ hospital for 8 days. All the tests and X-ray were done. I was given medicines for TB. I took medicines for almost a month. But I could not eat food after consuming medicines. I used to vomit those tablets. I had cough too. Then in the month of October, I was asked to go to the Government Centre. We spent nearly 5 months with the private clinics.”

(A 17-year- old female with Pulmonary MDR-TB)

**Reluctance for referral to RNTCP leading to out-of-pocket expenditure.**Some participants described a reluctance of their private providers to refer them early to the RNTCP. The RNTCP offers free TB diagnosis and treatment. Visits in the private sector incurred them significant out of pocket cost. This might be evident from a following narrative.

“Almost 1.5 years back, I started experiencing pain in my lower back. I went to a private doctor in Nigadi. I was given a medicine which relieved my pain. I then continued to have the medicines for 6 months, for which I spent around 8000 rupees ($93). My pain restarted, then I consulted another private doctor who asked me to get a CT scan done which costed me 3500 rupees ($50). I told the doctor—I cannot continue with the medicines because I cannot afford them. I asked him whether I can take the treatment from the Government health centre (RNTCP), but he told me that the Government treatment will not work. However, I visited the Government centre on the next day and started my treatment from there”.

(A 35-year-old male with extra pulmonary MDR-TB)

The above narratives point out a potential conflict of interest that private providers may have in referring patients to the RNTCP specifically the monetary gain of providing care in the private sector. Low suspicion and limited knowledge of extrapulmonary TB symptoms likely also contributed.

### Experiences within the RNTCP

Sixty-five (50%) participants reported that they had a prior history of TB and of them, 70% received treatment from the RNTCP for an earlier episode. When asked about their RNTCP experience, 28 (22%) participants reported that they were unsatisfied with the healthcare service. The following narratives provide insights to participants’ experiences with the RNTCP staff such as stigmatising behaviour, negligence toward patients’ problems, lack of hygiene in the referral centre for MDR-TB and vicinity with other patients who had drug resistant TB.

### Stigmatising or non-courteous behaviour

The following narratives illustrate patients’ frustration with the RNTCP and experience of stigmatizing behavior which resulted in treatment interruption.

“My tests were done in the private hospital, and I came to know that I had MDR-TB. I was started on treatment there itself for three days. But our family doctor advised us to go to the government centre for free treatment. So, we went to the government centre. However, after visiting there, I did not feel like going there because it was a dirty place. I had several questions in mind. But they were not talking to me. They were telling me to sit aside from them (stigmatising). I felt reluctant to go there for care".

(A 28-year-old female with pulmonary and extra pulmonary TB)

“I was having problems because of medicines. When I went to see the doctor, my face was already covered with the *burqa (Muslim women cover their face)*, but they asked- ‘why did you not cover your mouth? You have MDR-TB.’ I said my face is covered. Then they said lot of things. Even the sister (nurse) said a lot [unkind words] while giving injections. Though I tried to speak nicely, they were not speaking nicely. Then my father and husband spoke to the head doctor of the clinic about taking injections from our family doctor. The doctor did not pay attention. Then I stopped taking injections from that sister.”

(A 29-year-old female with pulmonary TB)

#### Unclean hospital environment and unwilling contact with other patients

Twenty three percent (29/128) participants expressed concerns about an unclean environment in the referral hospital where they were admitted for initiating MDR-TB treatment. It even adversely affected the food intake in some cases. A few of them also complained about unwilling contact with other (drug resistant) patients at the referral center.

“Since the first time I was taken there (in the government hospital), I did not feel like staying just by looking at the other patients. I told them to shift me somewhere else and not to keep in that hospital as it was not clean. I fought [with him] over this. I did not eat for three days though I was on medications. I wanted to complain [about the lack of hygiene] but what to do? I didn’t say anything.”

(A 29- year-old male with pulmonary TB)

“The hospital was unclean and other patients were also there. This disease can spread. My mother told the doctor that she would not admit me there. They gave me 17 tablets and I vomited all of them. There was no wash basin, so I had to go to the toilet which was dirty. I could not even stand there”.

(A 30-year-old female with pulmonary TB)

“There were 5–6 patients in the same room where I was admitted. There was a patient from a village. He used to cough and spit there on the floor. Looking at that other person, I did not feel like eating. I stayed like that for 3–4 days. Then my medications were started, and I was referred to a centre near my house”.

(A 30-Year-old male with pulmonary TB)

#### Problems faced due to injectables

Thirty-eight (30%) participants complained about swellings and bleeding at site where injection (Kanamycin) was administered by the staff. Some of them mentioned that they discontinued treatment for two or more weeks because of swelling as the staff used to inject at the same site despite the development of hematomas.

“Because of injections, I had swellings. I used to apply ice on that. The needle of the injection was long. The swellings became hard. I consulted a lady who provided massage for those swellings".

(A 30–year-old female with Pulmonary TB)

“I took injections for 3 months. A *‘gaath’* (bump/swelling) was formed at the site of injection so it became difficult to get additional injections at the point. But the doctor still used to give it there (at the same site). Then there used to be bleeding from that area and my cloths used to get wet.”

(A 28-year- old male with Pulmonary TB)

#### Barriers in receiving medicines

About 12% participants mentioned that they sometimes faced problems in getting medicines from the health centres. They reported following challenges-

“What happened, they used to give medicines for 3–4 days, and then when I go back, they used to say- ‘come tomorrow or a day after. We have no medicines in stock’. It was a wastage of time and again I had to go to the health centre.”

(A 15-year-old female with pulmonary TB)

“The centre is one km from our house. Two-three times they said medicines were not available. So, I had to travel further and get medicines from the other centre”.

(A 23-year-old female with pulmonary TB)

#### Depression and suicidal ideation

Sixty four percent participants reported symptoms that indicate depression due to TB, its long-term treatment, and experienced side-effects. Seven participants reported that they had thoughts of committing suicide and three of them attempted it.

“I thought it is better to die rather than take medicines every day. I tried to commit suicide twice. Once when nobody was at home, I tried to hang myself, but I suddenly felt choked up. So, I dropped the idea of hanging. I thought about how long my treatment was going to take. I then swallowed a few tablets, which I found at home. Those were not TB tablets. But nothing happened to me, though I experienced severe vomiting.”

(A 24-year-old female with pulmonary TB)

“I attempted suicide twice because I was very frustrated with the treatment and pain. Once I swallowed a lot of TB pills and second time jumped in the river.”

(A 41- year-old male with pulmonary TB)

## Discussion

To the best of our knowledge, this is the first qualitative research study in such large representative sample of individuals with MDR-TB from India. It presents patients’ firsthand experiences as they navigated MDR-TB care in the private sector and the RNTCP/NTEP. As mentioned by Haldane et al. 2021, delays in accessing health services can be dichotomized as related to patient factors limiting initial access to care, and to system factors after the patient’s first encounter to the health care system [14]. Our study explored the later one. Though India has a national TB control program for over three decades, this study shows that the private sector remains the first and main source of help-seeking for TB. Earlier studies by Uplekar and Shepard and by Udwadia et al. from India highlighted an ineludible role of the private sector in TB management. However, a significant number of private practitioners have prescribing practices that deviate from the standard TB treatment guidelines [15,16], likely driven by poor knowledge of MDR-TB treatment [16,17]. Another study by Das et al. in Delhi confirmed these findings using standardized TB patients [18]. Our recent study among 100 private practitioners in Pune city found that only 50% could identify the NAAT as the indicated point of care test to detect MDR-TB (unpublished). The observations from the present study add to these findings.

Prior anti-TB treatment is a major risk factor for MDR-TB [8,19]. However, an equal proportion of new and previously treated MDR-TB cases as observed in this study suggest ongoing transmission of MDR-TB strains in Pune city as reported in the quantitative findings [8], which is a serious concern. Poor knowledge of MDR-TB diagnosis and management among the general practitioners can delay diagnosis and eventually result in transmission of strains in the community from open cases of drug resistant TB [20]. It can also lead to amplification of resistance due to the blind practice of continuing the first-line regimen as reported by Kim and Famer [21]. Our study thus highlights a need for educating private practitioners in early suspicion of MDR-TB and importance of early referral of MDR-TB suspects for the NAAT (Xpert assay) at the NTEP centers. The NTEP officials can play a crucial role in conducting interactive sessions with private practitioners and help initiatives that strengthen public-private partnership. From a medical anthropology perspective, one needs to understand the dynamics of the Indian health system. It is an ecosystem of which patients, the NTEP and a large private sector, laboratories, and pharmacies are integral part [22]. Despite the implementation of mandatory TB notification policy since 2012, private sectors’ participation in TB control remains limited. Multiple strategies such as incentive-based models, public-private mix initiatives for including private sector in TB control have been suggested and successfully worked out in different countries [23,24]. However, considering the medico-pluralism (co-existence of different medical systems such as Allopathy, Ayurveda, homeopathy, and Unani), people’s behavior and the scale of the country, India will need diverse approaches for tackling MDR-TB. It is also important to understand the reluctance of the private sector to participate in TB control. There are issues of loss of their income, lack of timely updates on guidelines and feedback from the NTEP etc. The policy discussions need to be held at different levels with multiple stakeholders. Medical education needs to be revisited.

Apart from the private sector, participants expressed their non-satisfaction about the RNTCP/NTEP and brought to attention issues such as unhygienic environment in the referral center, and problems experienced while administering an injectable. Further, we identified that getting admitted in an inpatient TB ward for initiation of MDR-TB therapy was a concern for some participants as they expressed fear of catching serious infections from the surrounded patients. Provision of sputum cups for patients in the same ward/room can help to prevent the spread of infection that occurs due to spitting habits. Another issue was administering Kanamycin injection. It was partly a problem of the health staff’s lack of skills. However, with the updated guidelines, Kanamycin is removed from the regimen for MDR-TB as the absolute risk of adverse events (mainly hearing loss) was found to be high along with poor treatment outcomes [25]. Bedaquiline (a replacement for Kanamycin) based oral regimen was found to be with better outcomes [26]. With the policy change of including all oral regimen, patients may not face this injectable problem; nevertheless, in future if at all any injectable drug becomes a part of the regimen, the NTEP staff at health centers will need to be trained in administering it and in managing the side-effects. Our study further identified the need for facilities for offering care for psychological and/psychiatric conditions.

Farmer et al. argued that the ‘structural violence’ or external societal and organizational forces such as poverty, health policies and health system-related factors exist, which create barriers to care [12]. They further argued that although structural factors are beyond the control of patients, they may still be within the reach of medical and public health practitioners and frequently within the reach of policy makers. Our study is unique in addressing the structural violence in the form of unmet needs felt by the study participants themselves in an Indian setting-a country that carries the highest global burden on TB and DR-TB.

## Limitations

Our study has several limitations. First, since our study duration was only 18 months, we could not collect information about the entire course of treatment and outcomes for many participants. That limited our ability to provide insights into long-term treatment experiences. Second, we could not explore any gender specific unmet needs that we hope to address in future work. Third, we could only interview patients registered with the RNTCP/NTEP and could not address the needs of patients that are treated exclusively in the private sector.

## Conclusion

Using qualitative data on experiences of individuals with MDR-TB, we identified several unmet needs. We strongly believe that a careful attention toward fulfilling those needs would help improve the delivery of patient centered care and the treatment outcomes. Addressing the identified needs will also pave the path toward achieving Indian Government’s ambitious goal of TB elimination by 2025.
